# Real-Time Sensing of O-Phenylenediamine Oxidation on Gold Nanoparticles

**DOI:** 10.3390/s17030530

**Published:** 2017-03-07

**Authors:** Ru-Jia Yu, Jia-Jia Sun, Heng Song, Jing-Zhi Tian, Da-Wei Li, Yi-Tao Long

**Affiliations:** 1 Key Laboratory for Advanced Materials, Shanghai Key Laboratory of Functional Materials Chemistry & School of Chemistry and Molecular Engineering, East China University of Science and Technology, 130 Meilong Road, Shanghai 200237, China; yurujia@mail.ecust.edu.cn (R.-J.Y.); jiajiasun@mail.ecust.edu.cn (J.-J.S.); songheng@mail.ecust.edu.cn (H.S.); ytlong@ecust.edu.cn (Y.-T.L.); 2 College of Chemistry and Chemical Engineering, Qiqihar University, 42 Culture Road, Qiqihar 161006, China; tjz6666@163.com

**Keywords:** surface-enhanced Raman spectroscopy, oxidation of o-phenylenediamine, real-time monitoring

## Abstract

Real-time monitoring of chemical reactions is still challenging as well as important to study reaction mechanisms and reaction kinetics. Herein, we demonstrated the real-time monitoring of o-phenylenediamine (OPD) oxidation on the surface of gold nanoparticles by surface-enhanced Raman spectroscopy (SERS). The oxidation mechanism and the reaction kinetics were investigated on the basis of the SERS spectrum variation and the related density functionalized theory calculation. It was shown that the oxidation of OPD in the presence of copper ions was a two-step process of the deprotonation of the amino group on the aromatic rings and the rearrangement of the electron cloud to a π-conjugated system, which may open a new door to comprehensively understand the reaction process.

## 1. Introduction

Monitoring and characterizing chemical reactions is of vital significance to study the mechanisms and kinetics during the reactions [1]. For this purpose, many analytical methods have been used to investigate chemical reactions such as mass spectrometry, chromatography, localized surface Plasmon resonance, nuclear magnetic resonance spectroscopy, etc., while they generally can hardly provide in situ information on chemical reactions and have the disadvantages of limited sensitivity and slow response time. Surface-enhanced Raman spectroscopy (SERS) has been used to detect the vibration of molecules, providing finger-printing information. Due to the ultrasensitive detection, it also has been widely used as an analytical method [2,3,4,5]. In the 1990s, Zhang and co-workers performed fundamental works by using the SERS method to investigate the dynamic process in electrochemical reactions [6,7,8,9,10]. Recently, SERS has been employed for the in situ monitoring of the photochemistry of 4-nitrothiophenol [11] and for monitoring catalytic reactions on gold nanorods and the controlled release of the payload for gold nanocages [12,13,14]. Briefly, SERS has been used as a label-free method for studying reaction mechanisms [15,16,17,18,19].

O-phenylenediamine (OPD) is a well-known chromophore that is highly fluorescent and has been typically used to prepare various heterocyclic compounds such as benzimidazole, quinoxaline and benzotriazole, etc. The oxidation of OPD is extremely important since it has advantages in preparing conductive polymers [20,21] and in synthesizing carbon dots [22,23]. The colorless OPD can be oxidized into the fluorescent product OPDox, which develops a yellow color in solution. Based on the turn-on fluorescence and distinct color change in solution, the oxidation process of OPD has been widely used as a chromogenic substrate for horseradish peroxidase and has extensive application in immunoassays. Moreover, the oxidation of OPD in the presence of copper ions, silver ions and hydrogen peroxide has often been used to construct various biosensors [24,25,26,27]. Therefore the study of the oxidation process of OPD is of profound guiding significance, especially for constructing chemicals and biosensors. However, due to the lack of a real-time analytical method, clearly understanding the OPD oxidation mechanism and reaction kinetics is still challenging.

**Scheme 1:**
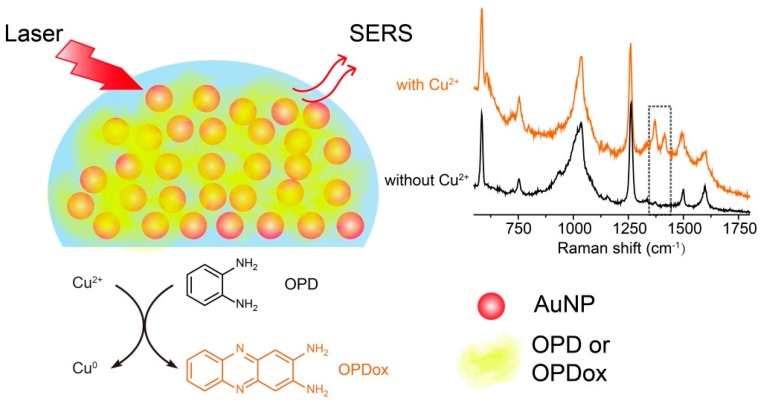
Schematic representation of the real-time monitoring of OPD oxidation on the surface of gold nanoparticles.

To study the reaction kinetics and oxidation mechanism of OPD, the reaction between OPD and copper ions was employed as our model. Herein, by using SERS spectroscopy, the real-time monitoring of the reaction mechanism and the reaction kinetics were investigated on the surface of gold nanoparticles (AuNPs) (Scheme 1). The molecular structure of the target OPD on the surface of AuNPs was easily observed with high time resolution. The employment of the SERS methodology is expected to provide fingerprint signals of potential intermediates and to detail the oxidation process.

## 2. Materials and Methods

### 2.1. Reagents and Instruments

The reagents used in this experiment were of analytical grade and used without any purification. O-phenylenediamine (OPD) and CuCl_2_·2H_2_O were purchased from Aladdin (Shanghai, China). HAuCl_4_·3H_2_O, trisodium citrate and 2,3-diaminophenazine were obtained from Sigma-Aldrich (St. Louis, MO, USA). Ultrapure water (18 MΩ·cm) from Millipore system was used in the preparation of all solutions. UV-Vis spectra were acquired on an Ocean optical USB 2000+; Raman spectra were measured with a portable Raman spectrometer (BWS415, B&WTEK, Newark, DE, USA) equipped with a 785 laser. The diameter and morphology of gold nanoparticles were characterized with a transmission electron microscopy (TEM, JEOL, JEM-2100, Tokyo, Japan) which was applied at a voltage of 200 kV.

### 2.2. Synthesis of AuNPs

AuNPs were prepared as previously reported [28]. In a 100 mL round bottom flask, 50 mL of 0.048 wt % HAuCl_4_ was heated to rolling boil to reflux with vigorous stirring. 5 mL of 1.0 wt % sodium citrate was added dropwise and the mixed solution changed from colourless to dark red. The resulting solution was kept boiling for 15 min after the color stay stable. The final solution was cooled to room temperature and characterized by UV-Vis spectrometer and TEM.

### 2.3. SERS Spectra of OPD

The synthesized AuNPs were condensed by centrifugation for 10 min at 5000 rpm and re-dissolved in ultrapure water. The condensed AuNPs were added to OPD solution (10 µM) and mixed thoroughly for 10 min. Copper chloride was added to the mixed solution of AuNPs and OPD and the final concentration of copper was 10 µM. Final solution was incubated at 37 °C according to previous studies [26,27] and SERS spectra were immediately measured at the mixed time of 0, 1, 5, 10, 20, 30, 60, 120, 180 min, respectively.

### 2.4. DFT Calculation

To further understand the oxidation of OPD, DFT (density functional theory) calculation was performed to determine the chemical structures and stimulate Raman spectra by using Gaussian 09 suite of program at the level of B3LYP/6-311++g(d,p) [29]. Considering that the reaction could proceed in aqueous solution, the IEF version of the PCM was included to optimize the calculation of geometry structure and vibration frequency [30]. Raman shifts were calculated on the basis of the vibrational frequency by 0.9637 [31], which was one of the basic settings.

## 3. Results and Discussion

**Figure 1:**
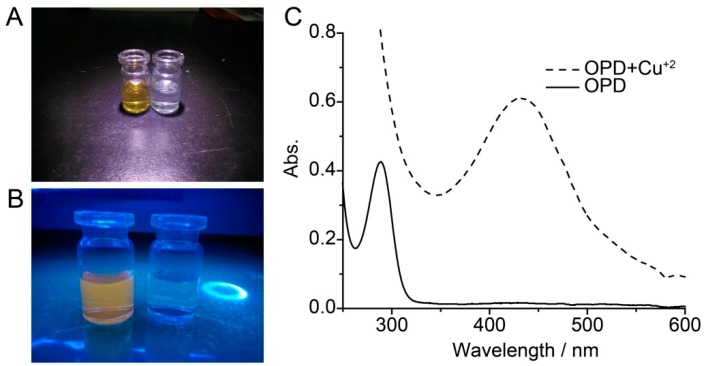
The characterization of the reaction of OPD in the presence of Cu^2+^. (**A**) A photograph showing the OPD solution in the presence (left) and absence of (right) Cu^2+^; (**B**) Florescent photograph of the above solutions under irradiation by 365 nm ultraviolet light; (**C**) UV-Vis spectra of the solutions.

The chemical reaction of OPD in the presence of copper ions was firstly demonstrated in Figure 1. The colorless OPD solution turned to yellow after 2 h incubation with Cu^2+^ (Figure 1A). This was followed by the significant enhancement of the fluorescence with irradiation by 365 nm ultraviolet light, as demonstrated in Figure 1B. Strong orange fluorescence emerged in the solution after the addition of Cu^2+^. This reaction could further be verified by the UV-Vis spectra in Figure 1C. The absorbance peak of OPD at 289 nm was red-shifted to 433 nm and the absorbance intensity was greatly increased after the reaction, which is in agreement with previously reported results [32]. The reaction of OPD in the presence of Cu^2+^ could thus be verified, but the reaction process was still unclear due to the limitation of the characteristic methods.

**Figure 2:**
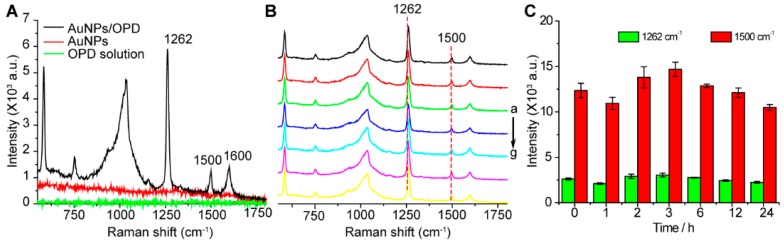
(**A**) SERS spectra of the AuNPs/OPD, AuNPs and OPD solutions; (**B**) Raman response of the AuNPs/OPD solution under incubation at 37 °C for (a) 0 h, (b) 1 h, (c) 2 h, (d) 3 h, (e) 6 h, (f) 12 h, (g) 24 h; (**C**) SERS intensities at the bands of 1500 cm^−1^ and 1262 cm^−1^ plotted with different incubation times. Error bars were based on three independent measurements.

To investigate the reaction mechanism of OPD, this reaction was monitored in real time using the proposed SERS method. Gold colloids were prepared by the sodium citrate reduction of HAuCl_4_ as previously described [33]. The gold nanoparticles were characterized by UV-Vis spectra (Figure S1a) and transmission microscope (TEM) images (Figure S1b) with a diameter of 60 nm. The synthesized gold colloids were mixed with OPD solution to obtain the SERS spectra of OPD. As shown in Figure 2A, the characteristic SERS spectrum was only presented in the presence of both gold nanoparticles and OPD solutions. The Raman band at 1500 cm^−1^ was attributed to the stretching and in-plane bending of the C=C bond in the benzene ring, while the peak at 1262 cm^−1^ corresponded to the stretching vibration of the C–NH_2_ bond. Thus the Raman bands at 1262 cm^−1^ and 1500 cm^−1^ would be regarded as characteristic peaks of the OPD molecule [34]. Further, the SERS method could be used to characterize the OPD molecules and then could be utilized to investigate the reaction mechanism. The stability of the SERS spectrum of OPD was also investigated. SERS spectra of the mixed solution with different incubation times are depicted in Figure 2B. The SERS intensities at 1500 cm^−1^ and 1262 cm^−1^ remained nearly stable when incubated at 37 °C for 24 h (Figure 2C). The stable SERS spectrum of OPD indicates that the variation of the spectra could only be caused by changes of the OPD molecules in the next experiment.

**Scheme 2:**
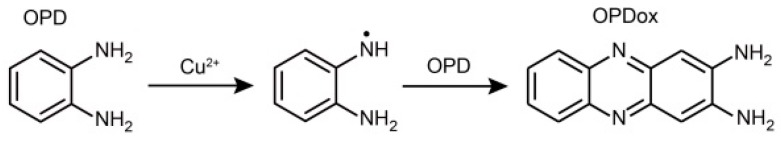
The proposed mechanism of the oxidation of OPD in the presence of copper ions.

**Figure 3:**
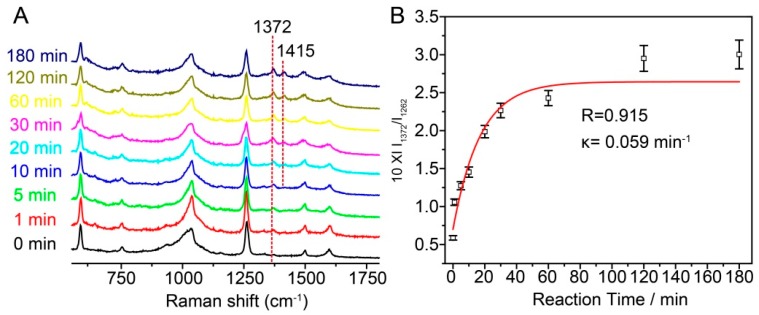
(**A**) SERS spectra of AuNPs/OPD solution in the presence of Cu^2+^ at different reaction times under incubation at 37 °C; (**B**) Plot of 10 × I_1372_/I_1262_ against different reaction times.

The real-time monitoring of the reaction of OPD in the presence of Cu^2+^ was then performed using SERS spectra. An excess concentration of Cu^2+^ was added to the mixed solution of gold nanoparticles and OPD. Kinetic monitoring of the chemical reaction process was implemented by recording SERS spectra at different reaction times. Successive Raman spectra were recorded until no noticeable difference was found between the adjacent spectra. Based on the measured SERS spectra and the corresponding DFT (density functional theory) calculation (shown in Table S1), a fundamental understanding of the OPD oxidation by Cu^2+^ was observed. As shown in Figure 3A, after the addition of Cu^2+^, the Raman band at 1372 cm^−1^ occurred immediately which could be attributed to the C=N–C stretching mixed with ring stretching. The value of I_1372_/I_1262_ increased rapidly as the reaction progressed. Meanwhile, the Raman band at 1415 cm^−1^, assigned to the in-plane bending of the C=N–C bond, appeared 20 min later. The generation of the C=N bond may demonstrate the oxidation of OPD to generate OPDox (2,3-diaminophenazine) on the surface of the AuNPs. Moreover, the reaction product has a SERS spectrum highly similar to that of pure 2,3-diaminophenazine (Figure S2), which further indicates the production of OPDox. It could be estimated that the oxidation of OPD with Cu^2+^ may be a two-step process, as demonstrated in Scheme 2. Firstly, the amino group on the OPD molecule deprotonated to form an imide radical, which could substitute para-phenyl, and then the electron cloud of the molecule rearranged to a C=N-conjugated system.

This oxidation of OPD in the presence of Cu^2+^ observed by SERS method could also be confirmed by high-resolution electrospray ionization mass spectrometry (HREIMS). The sample was prepared using the mixed solution of AuNPs/OPD after 30 min incubation with Cu^2+^ under 37 °C. As demonstrated in Figure S2, the mass spectrometry results demonstrated a new mass peak at *m*/*z* 210.0905 besides the original OPD peak, which convincingly demonstrated the generation of the oxidation product (OPDox) on the surface of AuNPs in the presence of Cu^2+^. It matched well with the appearance of the benzene ring breath vibration and the C=N stretching vibration band at 1372 cm^−1^ and 1415 cm^−1^, respectively. Therefore, the reaction process demonstrated by SERS was verified by conventional mass spectrometry. Comparing these two technologies for reaction monitoring, SERS could monitor the reaction in real time, provide fingerprint information of the reaction intermediates and test trace amounts of analyte. In brief, a detailed reaction process could be obtained through the SERS approach and it is superior for reaction monitoring in real time.

To further acquire the kinetic parameters of the oxidation reaction, the intensity ratio of I_1372_/I_1262_ was plotted with different reaction times, as shown in Figure 3B. We observed a time-dependent exponential increase of the intensity ratio within 180 min. Since excess Cu^2+^ was used, the reaction for the oxidation of OPD in the presence of Cu^2+^ could be presumed to follow pseudo-first-order kinetics. The reaction rate constant κ can be calculated by Equation.

(1) $$\kappa t=\ln\frac{{\left(I_{1372}/I_{1262}\right)}_{t=0}}{{\left(I_{1372}/I_{1262}\right)}_{t}}$$

Here, (I_1372_/I_1262_)_t=0_ is the intensity ratio of these two Raman bands at the beginning of the reaction and the (I_1372_/I_1262_)_t_ is the intensity ratio at time t. It can be calculated that the rate constant κ for the oxidation of OPD in the presence of Cu^2+^ is 0.059 min^−1^ (Figure 3B).

## 4. Conclusions

In conclusion, the monitoring of the oxidation of OPD on the surface of AuNPs in the presence of Cu^2+^ was performed by using the SERS strategy. The results suggested that the oxidation of OPD on the AuNP surface was a two-step process: the amino group on the aromatic rings firstly deprotonated and attacked the para-position of another OPD molecule, and then the electron cloud of the whole molecule rearranged to a π-conjugated system. The reaction kinetics was further determined from the reaction time-dependent SERS intensity related to the oxidative process of OPD. Moreover, due to the ability of providing information on the intermediate state of the reaction, SERS offered advantages over other analytical methods such as UV-Vis spectra, fluorescence spectra and HREIMS in the real-time monitoring of the reaction, showing promise for comprehensively understanding the reaction mechanism and reaction kinetics.

## Supplementary Materials

The following are available online at http://www.mdpi.com/1424-8220/17/3/530/s1. Figure S1: UV-Vis spectra (a) and transmission electron microscope image (b) of the synthesized gold nanoparticles with a diameter of 60 nm. Figure S2. SERS spectra of pure 2,3-diaminophenazine solution and AuNPs/OPD+Cu^2+^ incubated at 37 °C for 30 min. Figure S3. Mass spectrum (HREI^+^) of AuNPs/OPD+Cu^2+^ incubated at 37 °C for 30 min. Table S1: SERS Spectral data of AuNPs/OPD before and after reaction with Cu^2+^ and Raman spectral data calculated with DFT for OPD and OPDox corresponding band assignments.
