# Supplementary material for: Real-Time Sensing of O-Phenylenediamine Oxidation on Gold Nanoparticles
Source: Sensors (Basel). 2017 Mar 7;17(3):530. doi: 10.3390/s17030530 (PMC5375816; doi:10.3390/s17030530)
Supplement: Supplementary file 1 [file sensors-17-00530-s001.pdf]

Supporting Information

Real-time sensing of o-phenylenediamine oxidation on gold nanoparticles

Ru-Jia Yu[1], Jia-Jia Sun[1], Heng Song[1], Jing-Zhi Tian[2], Da-Wei Li[1], and Yi-Tao Long[1]*

[1] Key laboratory for Advanced Materials, Shanghai Key Laboratory of Functional Materials Chemistry, & College of Chemistry and Molecular Engineering, East China University of Science and Technology, 130 Meilong Road, Shanghai, 200237, P. R. China

[2] College of Chemistry and Chemical Engineering, Qiqihar University, 42 Culture Road, Qiqihar, 161006, P. R. China

* To whom correspondence should be addressed. E-mail: daweili@ecust.edu.cn; Tel/Fax: +086-21-6425-0032.

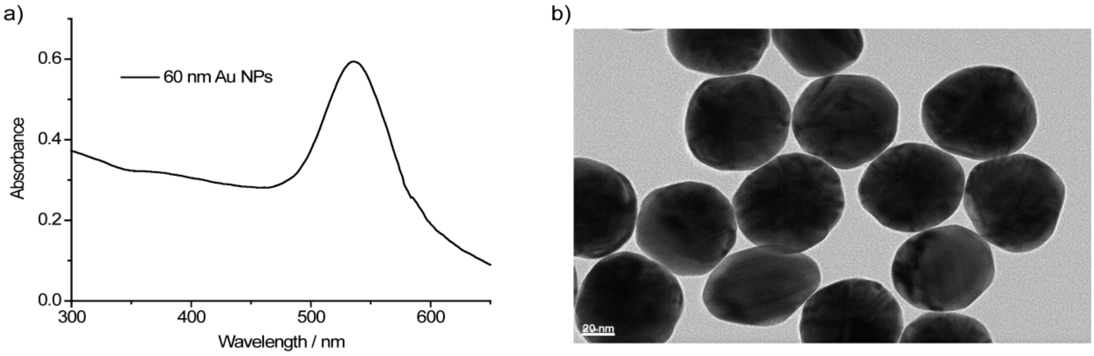

Figure S1. UV-Vis spectra (**a**) and transmission electron microscope image (**b**) of the synthesized gold nanoparticles with a diameter of 60 nm.

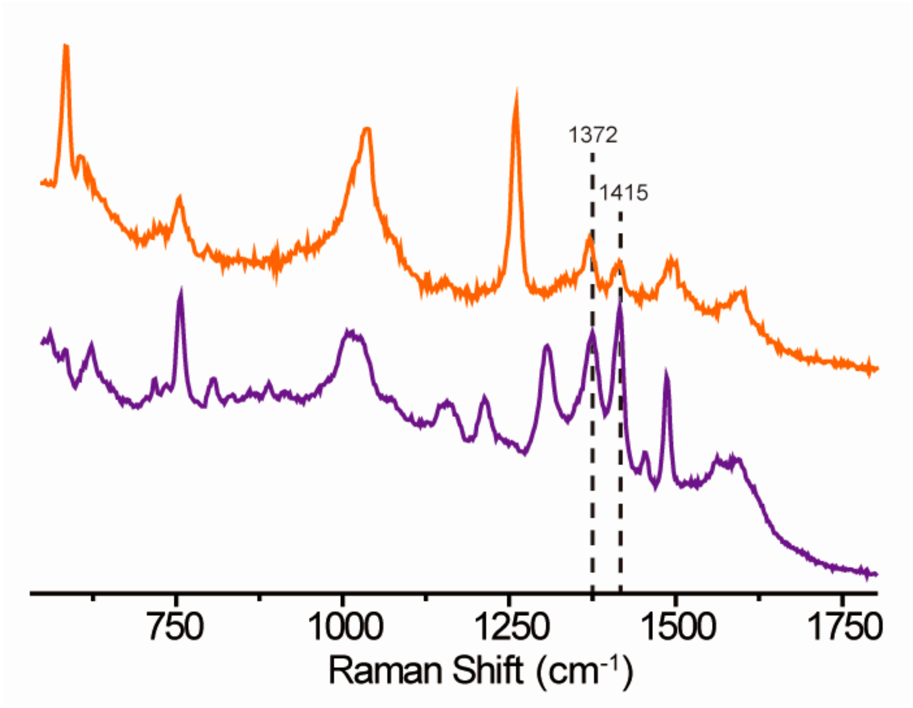

Figure S2. SERS spectra of pure 2,3-diaminophenazine solution (purple) and AuNPs/OPD+Cu^{2+} incubated at 37 °C for 30 min (orange).

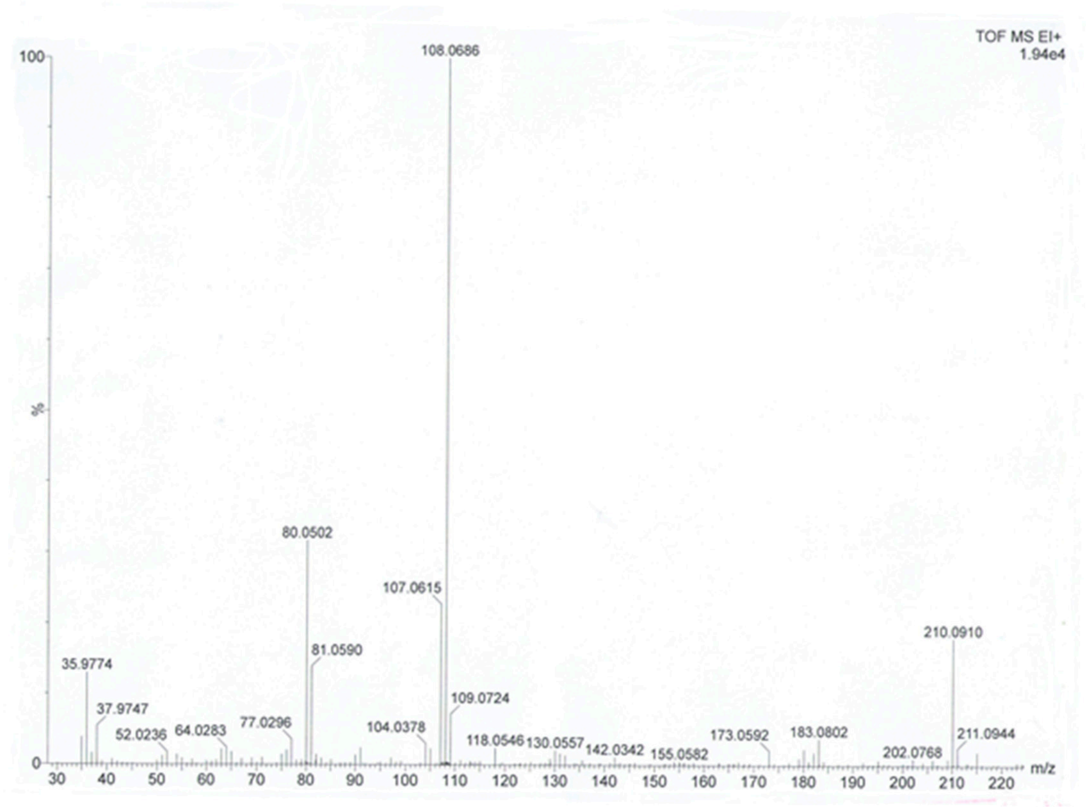

Figure S3. Mass spectrum ($HREI^+$) of Au NPs/OPD+Cu^{2+} incubated at 37 °C for 30 min.

Table S1. SERS Spectral data of AuNPs/OPD before and after reaction with Cu^{2+} and Raman spectral data calculated with DFT for OPD and OPDox corresponding band assignments.

OPD			OPDox		
Theory (cm^{-1})	Experimental (cm^{-1})	Assignment	Theory (cm^{-1})	Experimental (cm^{-1})	Assignment
592	585	ρCCC ring	586	585	ρCCC ring
760	753	υCC ring, υC-NH_2	752	753	υCC ring, υC-NH_2
1055	1037	υCC ring, δCCH ring	1034	1037	υCC ring, δCCH ring
1257	1262	δCCH ring, υCC ring, υC-NH_2	1262	1262	δCCH ring, υCC ring, υC-NH_2
1512	1500	υCCC ring, δCCH ring,	1372	1372	υC-N=C, υCC ring

		υC-NH_2, ρNH_2			
1592	1600	υCCC ring, δCCH ring, ρNH_2	1413	1415	δC-N=C
			1499	1500	υCCC ring, δCCH ring, υC-NH_2, ρNH_2
			1601	1600	υCCC ring, δCCH ring, ρNH_2

υ, stretching; δ, in-plane bending; ρ, scissoring
